# Monthly Summary

**Published:** 1877-10

**Authors:** 


### MONTHLY SUMMARY.

---

*The Question of Rest during Menstruation.*—Mary P. Jacobi, M. D., Prof. of Materia Medica, Women's Medical College, N. Y., in a recent work on the above subject, says: Judging by statistics we should say that immunity from menstrual suffering was to be expected in proportion to:

"1. The vigor of health during childhood and the soundness of family history, especially in regard to freedom from constitutional taint of scrofula, consumption, or rheumatism, or family tendency to uterine disease.

2. To the degree of exercise taken during school life.

3. To the thoroughness and extension of the mental education.

4. To the degree to which general health and capacity for exercise were maintained after cessation of study.

5. To steadiness of occupation.

6. Finally to marriage at a suitable time."

"When persons in good health suffer severe menstrual pain, this is nearly always dependent upon some anatomical imperfection of the uterus. When persons in delicate health are free from menstrual distress it is because the supplemental wave of nutrition does not rise sufficiently to greatly increase general vascular tension, or because the uterus is too slightly hyperæmiated to excite reaction." "Whenever women exhibit mental irritability and consequent weakness at, or before, menstruation, it is a proof that the resistance of their nerve centres is weakened, below the normal standard, sometimes congenitally and by inheritance. In these cases rest is desirable during whatever period of the month the nervous excitement may be experienced, but this will be more frequently through the two or three days preceding the hemorrhage, than at the time of the flow. In slighter cases rest is unnecessary; in those more severe it is in itself useless, i. e. other measures must be taken in order to cure the disease. In those cases of congenital hysteria where a cure can not be effected, it is evident that the women *are* permanently unfit to bear severe responsibility or to undergo mental strain." M. B. M.

---

*Simple Remedy to Stop Bleeding.*—Mr. F. E. Forster, of this city, informs us that lately he cut himself, and trying to stop the bleeding, he did not succeed, notwithstanding he tried to do it in several ways; finally the idea struck him to put on some dry plaster of Paris, which happened to be at hand. It stopped the bleeding at once, while it only caused some stinging sensation, lasting a minute or two, but no ill effects were experienced. He requests us to publish this for the benefit of others, and we do so cheerfully, thanking him for the good example he has set by giving this communication.—*Druggists Circular.*

---

*The Perils of the Cigar.*—Of course there are plenty of men who believe that smoking is positively injurious. We have had it proved over and over again that the man who smokes is sure to die. Even the living smokers who have by mistake grown old cannot dodge this dreadful and indisputable issue. Then again, before they die, these smokers are said to be addicted to loss of memory (forgetting their promises to reform, no doubt,) lack of will power (to stop the habit perhaps,) loss of sight, loss of muscular power, taste, smell, feeling, procreative power, and finally idiocy. All this is dreadful and causes us to rejoice that, for a time at least, we have given up the use of tobacco. But there is really a serious aspect to this matter which deserves more than ordinary attention, and that is the liability of syphilitic contamination through the medium of the weed. Recently an English journal cited the case of a young girl, a manufacturer of cigars, who with a chancre upon her lip moistened with saliva the tips of all the cigars she made for a period of three days. This is a well-authenticated case, and it is impossible to say how many more there are like it which are constantly spreading the dreadful poison to unsuspecting pleasure-seekers. The abominably disgusting practice among tobacco-strippers of moisting the leaf with spray from the mouth is doubtless an occasional, if not a common cause of infection.—*Med. Record.*

---

*Hydrobromic Ether as an Anæsthetic.*—Rabuteau, in a memoir read before the Academie des Sciences, states that he has investigated the physiological properties and mode of elimination of hydrobromic ether. He has satisfied himself that this anæsthetic agent, which possesses properties intermediate to those of chloroform, bromoform, and ether might be advantageously employed to produce surgical anæsthesia. The hydrobromic ether is neither a caustic nor an irrritant. It can be ingested without difficulty, and applied without danger, not only to the skin but to the external auditory meatus and to the mucous membrane. It is eliminated completely, or almost completely, by the respiratory passages, in whatever way it may have been introduced into the system.—*Lancet.*

---

*The Percussion of Bones.*—Percussion of bones is employed, either to find out the painful point in bones or to diagnose, by the character of the note produced, the pathological changes that have taken place in the bone. Lucke says he has made use of percussion only in the long bones, and has arrived at the following results: The epiphysis gives a higher note than the diaphysis. The corresponding bones of each side give out the same note. Freshly united fractures give out a deeper note. Cronic central ostitis gives a deeper note than normal. In a case of chronic gonitis the diaphysis of the affected tibia, which was porous, gave out a much higher note than the opposite healthy one. The percussion had better be performed with the extremities hanging, and in the lower extremities not touching the ground.—(Prof. Lucke, quoted in *Schmidt's Jahrtuche.*

---

*On Ranula.*—In a contribution [*Gazette Hebdomadaire,* No. 16, 1877,] giving short clinical reports of six cases of ranula observed by himself, Prof. Michel, of Nancy, discusses the nature and situation and the surgical treatment of this form of new growth. In each of these cases excision of the cyst was practised with complete success. From observations made during these six operations, and also from dissection of a ranula in a dead subject, the author has been convinced that in the majority of instances of this affection, the cyst in its development has no connection with any of the salivary ducts. The view that ranula may be due to dilatation of the ducts of the sublingual or submaxillary glands is not altogether rejected ; but it is held that, in the majority of cases, the cyst has some other seat of origin. In all the seven specimens examined by the author there was an absence of any connection between the cyst and the salivary canals, and in each case the tumour had evidently originated in the areolæ of the connective-tissue about the frenum of the tongue. The so-called capsule of Fleischmann, fluid distension of which is supposed by Tilaux and other French surgeons to constitute ranula consists, according to Prof. Michel in nothing more than an occasional and abnormal dilatation of one or more of the areolæ of the sublingual connective tissue. On microscopical examination of the contents of the cyst in the above-mentioned seven cases, tesselated epithelium and crystals of cholesterin were found in some, and globular epithelium in others. In no specimen was the author able to obtain a reaction resembling that produced by saliva. Prof. Michel holds that expiration by the knife ought to be regarded as the general method of treatment for ranula ; and he argues that this proceeding first recommended by Heister, is free from many of the objections that have been raised against it by Sedillot. Far from being an impracticable operation in ordinary cases of ranula, it may, even in cases of severity and long duration, be readily and safely performed. Excision, though more difficult than the usual methods of surgical treatment, such as injection of iodine, batrachosioplasy and incision and cauterization combined, is attended with speedy as well as with most permanent results. No relapse had occured in any of the six cases treated by the author, five of which have been under his observation from time to time during many years. Two methods of extirpation are mentioned ; in one, the ranula is first freely incised and the walls of the emptied cyst then dissected away ; in the other the cyst is removed intact, together with its contents. The choice between one and the other of these methods should be guided by the thickness of the cyst-walls. When this wall is thin, preliminary incision is to be preferred ; when it is thick extirpation without incision should be practised.—*Brit. and Foreign Med.-Chir. Review.*

---

*On Lipoma of the Tongue.*—Tizzoni and Parona describe in the *Annali Universali di Medicina e di Chirurgia* for March, the case of Professor C. Gianni, aged 74, who had had a growth about a year, situated under the anterior and under surface of the tongue, on the right side. When he had had it six months it was the size of a filbert, and, thinking, it was a collection of pus, he punctured it himself, but let out only a little blood.

The tumour then rapidly grew, and attained the size of a large walnut, becoming very inconvenient, and causing constant spitting of saliva. Removal was effected by enucleation, and the nature of the growth was ascertained to be that of an ordinary soft lipoma. Microscopic examination confirmed the naked eye opinion. The patient did well. A *resume* is given of all the recorded cases of lipoma linguæ.—*London Med. Record.*

---

*To Remove the Smell of Carbolic Acid.*—It is recommended to rub the hands with damp flaxseed meal. The same substance it is said, will remove other odors.

---

*Chloral.*—Dr. Oscar Liebriech charges that American chloral is especially badly prepared. It should not be in cakes, but crystals.

---

*Doctors of Medicine.*—The medical colleges of this country graduated this year 1442 students. Truly might Abertheny's question be repeated : " Young gentlemen, what is to become of you all ?"

---

*Cause of the Breaking Down of Teeth in Gestation.*—Dr. J. Williams, Ohio State Dental Society, in a paper on the treatment of teeth during gestation and lactation, thinks that the primary causes of the breaking down of the teeth at these periods is due to " the premature age at which childbearing is commenced ; systemic unfitness for the reproductive function from other causes, and mental infelicity."

---

*Nitrate of Silver and its Specific Discoloration of the Skin.*—Prof. Pepper, (College of Physicians, Philadelphia,) calls attention to the fact that the earliest sign of the peculiar effect of nitrate of silver—the staining of the skin,—" argyria," is a peculiar blue line on the gums. The hue is darker and more decidedly blue than the " lead line," and so may be diagnosed. The salts of copper, it is stated, will produce a similar line on the gums.

